# Building resilience: analysis of health care leaders’ perspectives on the Covid-19 response in Region Stockholm

**DOI:** 10.1186/s12913-024-10886-4

**Published:** 2024-04-02

**Authors:** Carl Savage, Leonard Tragl, Moa Malmqvist Castillo, Louisa Azizi, Henna Hasson, Carl Johan Sundberg, Pamela Mazzocato

**Affiliations:** 1 Department of Learning, Informatics, Management and Ethics, Medical Management Centre, Karolinska Institutet, Tomtebodavägen, 18A, 171 77 Stockholm, Sweden; 2 Unit for Implementation and Evaluation, Center for Epidemiology and Community Medicine, Region Stockholm, Sweden; 3 Department of Physiology and Pharmacology, Karolinska Institutet, Stockholm, Sweden; 4 Södertälje Hospital, Södertälje, Sweden

**Keywords:** Covid-19, Organizational resilience, Leaders, Crisis leadership, Health care management

## Abstract

**Background:**

The Covid-19 pandemic has tested health care organizations worldwide. Responses have demonstrated great variation and Sweden has been an outlier in terms of both strategy and how it was enacted, making it an interesting case for further study. The aim of this study was to explore how health care leaders experienced the challenges and responses that emerged during the initial wave of the Covid-19 pandemic, and to analyze these experiences through an organizational resilience lens.

**Methods:**

A qualitative interview study with 12 senior staff members who worked directly with or supervised pandemic efforts. Transcripts were analyzed using traditional content analysis and the codes directed to the Integrated Resilience Attributes Framework to understand what contributed to or hindered organizational resilience, i.e. how organizations achieve their goals by utilizing existing resources during crises.

**Results/Findings:**

Organizational resilience was found at the micro (situated) and meso (structural) system levels as individuals and organizations dealt with acute shortages and were forced to rapidly adapt through individual sacrifices, resource management, process management, and communications and relational capacity. Poor systemic resilience related to misaligned responses and a lack of learning from previous experiences, negatively impacted the anticipatory phase and placed greater pressure on individuals and organizations to respond. Conventional crisis leadership could hamper innovation, further cement chronic challenges, and generate a moral tension between centralized directives and clinical microsystem experiences.

**Conclusions:**

The pandemic tested the resilience of the health care system, placing undue pressure on micro and meso systems responses. With improved learning capabilities, some of this pressure may be mitigated as it could raise the anticipatory resilience potential, i.e. with better health systems learning, we may need fewer heroes. How crisis leadership could better align decision-making with frontline needs and temper short-term acute needs with a longer-term infinite mindset is worth further study.

**Supplementary Information:**

The online version contains supplementary material available at 10.1186/s12913-024-10886-4.

## Introduction

The Covid-19 pandemic tested health care organizations worldwide [1–5]. The rapid spread and high patient volumes bore with them a continual threat to overrun health systems, particularly within acute care [6]. Shortages in personal protective equipment (PPE), medications, ventilators, ICU and ward beds, staff, and morgue space were commonplace [7]. Staff experienced extraordinary physical and emotional demands, compounded by treatment uncertainties, ethical dilemmas, fear of becoming infected or infecting others [8]. Altogether, the challenges to health systems were multiple, urgent, and very real.

A pandemic is an unexpected disturbance of daily health care operations and can be described as a “low-chance, high-impact” (albeit inevitable and recurring) event [9, 10]. In these situations of volatility, uncertainty, complexity, and ambiguity, research suggests that even our best-laid schemes (often) go awry [11, 12]. Instead, health systems need to be resilient, agile, and improvise to respond effectively by increasing autonomy, maintaining structure, and creating a shared understanding [13]. Therefore, organizational resilience may provide a relevant framework to understand the response of health care organizations to the pandemic [13].

Many organizations demonstrated the capability to innovate, partner with others, develop virtual care solutions, e.g. video and phone consultations, eVisits, eConsults, and chatbot messaging– all of which have seen widespread adoption [14, 15]. Others became frustrated by the slow response to acute challenges and perceived a lack of coherence in resource supply chains, recommendations, and treatment guidelines. Global uncertainties were addressed by local actions as managers and health care professionals took extraordinary measures to solve problems. As these examples illustrate, and Ashby’s “Law” of Requisite Variety dictates, low-chance, high-impact events require multi-level responses that are of a matching level of complexity [16]. Thus, local health care solutions are impacted by national public health and political strategies, which in turn are often dependent on international decisions.

Sweden has been an outlier in its choice of response (fewer mandatory restrictions) and timing (often later) in comparison with many other countries, even if physical mobility and social behavioral patterns were similar [17–19]. For example, lockdowns were avoided, far fewer restrictions were introduced, and they were less restrictive. The focus was on voluntary compliance to the governmental recommendations of social distancing, working from home, and mask use in crowded areas. Adherence to guidelines was high. Covid passes were only introduced for travel and there were no restrictions on outdoor activities. These national strategies themselves are worth studying, as are the strategies of Swedish health care providers working in this context. Therefore, the aim of this study was to explore how health care leaders experienced the challenges and responses that emerged during the initial wave of the Covid-19 pandemic, and to analyze these experiences through an organizational resilience lens.

## Methods

### Study design

We employed a qualitative interview study design to inductively explore the challenges and responses. Upon reviewing the findings, we identified patterns that could be further analyzed through a resilience lens. We therefore directed the empirical findings to the Integrated Resilience Attributes Framework to abduct, i.e. logically infer, how they contributed to organizational resilience [20, 21].

### Theoretical framework

Resilience refers to the ability to utilize existing resources in the face of a crisis [22]. It can be seen as an organization’s ability to recover and regain its original functions after a period of stress. Through the advancement and development of processes and capabilities, the organization can exit the crisis stronger than before. A key component is predictive planning, i.e. the ability to anticipate the arrival of a crisis. This suggests that resilience is more than merely a defensive posturing that promotes survival, but an active response that engenders development.

Organizational resilience can be conceptualized as consisting of spatial and temporal moments [23]. It is located at different levels, i.e. the micro, meso, and macro (system) levels. These can be described as situated, structural, and system versions of resilience. Situated resilience involves the (immediate) use and combination of existing socio-technical resources. Structural resilience involves a mid-term (weeks to years) deliberate restructuring and redesign of socio-technical resources. Systemic resilience involves a long-term overhaul of how socio-technical resources are structured and utilized [23]. Building upon Hollnagel [24], resilience can be conceptualized as involving iterative cycles of behaviors related to anticipation, monitoring, responding, and learning at the micro, meso, and macro levels (Anderson et al., 2020). Anticipation involves identification of critical developments and potential threats. Monitoring involves keeping tabs on internal and external developments. This allows responding, which involves acceptance of the new reality created by the crisis; and coordinated, collective sense-making, and fast-responses to develop and implement new solutions. Learning, i.e. adaptation, involves emerging from a crisis in a stronger form through capabilities related to reflection and learning and organizational change. Learning involves first incorporating the lessons learned into the existing knowledge base that leads to change through the inception of new norms, values, and practices, so-called second-order learning. The organization’s capability to effect change is thus a critical capability [25]. This combined view of organizational resilience is captured in the Integrated Resilience Attributes Framework (Anderson et al., 2020).

### Study setting

The Swedish health care system is highly decentralized with twenty-one self-governing regions responsible for funding and provision of health services [26]. Region Stockholm is the largest of these, with three acute teaching hospitals, one integrated acute and community care hospital, one eye hospital, and one university hospital (Karolinska University Hospital) serving a population of ca. 2 million inhabitants. A purchaser-provider model allows for patient choice and for private and publicly owned actors to work within the publicly financed system [27]. The majority of primary care, advanced home healthcare, as well as several outpatient specialist clinics are integrated into one provider organization, i.e. Stockholm Health Care Services (SLSO) [28]. Municipalities are responsible for care of elderly and disabled [26]. As in many health systems, challenges have been identified in terms of crossing organizational boundaries [29], particularly between tertiary and primary, psychiatric, and municipal care.

The first case of SARS-CoV-2 was registered in Sweden on January 31, 2020. Stockholm was among the first regions to prepare for these patients and soon faced one of the largest and fastest growing patient populations in the country. On February 7, Region Stockholm formed an emergency management team (RSSL) following the NATO emergency model [30]. On March 20, the Region Stockholm government directed SLSO to coordinate all operations in both private and public primary and community care as well as regional municipalities [30]. On March 27, RSSL established a regional Command Center to centralize and strengthen the supply of PPEs to health care staff [31]. The Command Center was coordinated by Karolinska University Hospital.

### Participants

Through purposive sampling, we sought to interview experienced individuals in senior positions within regional health care-oriented organizations who had played pivotal leadership roles in addressing the challenges the pandemic created (Table 1). These individuals had gained visibility either through the media or through internal communication channels. We also employed a snowball technique where participants were asked to recommend others to interview. Despite the pressure of an ongoing pandemic, we interviewed twelve participants (seven women and five men). All but two had backgrounds in medicine (anesthesiology, surgery, and internal medicine) or nursing. Most had management roles, and all worked directly with or supervised efforts to address the pandemic in intensive care units, emergency departments, wards, HR, or hospital management at six different hospital sites. Others supported these efforts through education, the regional crisis planning and response organization, or the medical technology industry.

**Table 1:** Characteristics of study participants (when interviewed)

Role	Professional Background	Type of Organization	Department/Clinic
Attending physician, head of research and education	MD, Attending, anesthesiology	Hospital	Intensive Care Unit
Head of medical training department and HR	RN	Hospital	Medical Training Center
Head of ICU	MD, Attending, anesthesiology	Hospital	Intensive Care Unit
Head of Nursing and Section Manager	RN	Hospital	Emergency Department
Section manager, Internal Medicine	MD, Attending, internal medicine	Hospital	Emergency Department
Education manager and nurse manager	RN	Hospital	Medical Training Center
CEO	Lawyer	MedTech	N/A
Operations Manager, Head of Geriatric Care, Stockholm Region	Administrator/ Management	Community health service	Crisis group
Operations Manager	MD, Attending, anaesthesiology	Hospital	Intensive Care Unit
Doctor, Medical Leader	MD, Attending, pediatrics	Hospital	Emergency Department
Operative Director	MD, Attending, plastic surgery	Hospital	Management
Assistant Manager	MD, Attending, anesthesiology	Hospital	Intensive Care Unit

### Data collection

Interviews were conducted between 28th of August 2020 and 21 of January 2021 using a semi-structured interview guide that allowed for follow-up questions (Additional File 1). The questions were arranged around five areas of inquiry: challenges, who was involved, responses, contextual factors, and lessons learned. The interview guide was pilot tested twice. Since no substantial changes were made, these interviews were included in the study. Interviews were mostly conducted online due to the pandemic; some at the place of employment. Most were approximately one hour in length, three were 45 min, and one 1h33 minutes long. They were digitally recorded and transcribed *verbatim*.

### Data analysis

Interview transcripts were read repeatedly to develop familiarity. Traditional (inductive) content analysis was performed using NVivo Qualitative Data Analysis Software; QSR International (2018) [32]. LT identified meaning units relevant to the research question and using their manifest meaning, summarized them as codes. To strengthen trustworthiness, all codes were reviewed by all authors, who then jointly sorted them into themes, categories, and sub-categories using the Miro online virtual whiteboard (www.miro.com). In a second analysis phase, the inductively derived categories and subcategories were directed to the Integrated Resilience Attributes framework and labelled as facilitating (+) or hindering (-) factors [20].

### Ethical considerations

Informed consent was sought and received prior to the interview. Data was handled confidentially, and all efforts were made to preserve anonymity. Participants were made aware that they could withdraw participation at any time. The Stockholm Regional Research Ethics Vetting Board formally stated that this research does not need an ethical permit.

## Results

An overview of the categories and sub-categories identified in the five areas of inquiry are presented in Additional File 2 and described in detail below.

### Poor preparation led to acute shortages

Challenges were associated with pandemic preparedness, acute shortages, barriers to response development, and the emerging consequences of being ill-prepared.

### Pandemic preparedness

Hospitals were described as unprepared for the pandemic, despite readily available knowledge about the pandemic and some staff with considerable international disaster relief experience.

The most distressing thing was that we knew this was coming. It didn’t come from nowhere. It was a catastrophic situation early in Italy. There were reports to colleagues warning us to prepare, “You have no idea what awaits.” We could have been more prepared. (P08)

Deficiencies in existing IT-systems led to the abandonment of digital innovations. Crisis leadership was faulted for unclear information, directives, and routines, e.g. conflicting infection control routines and large discrepancies between Swedish and WHO guidelines. Hospitals were better prepared than primary and elderly care and more able to repurpose wards and operating theaters to increase bed capacity.

### Acute shortages

Participants described acute shortages in personal protective and medical equipment, staff, and care capacity. Material shortages were linked to dismantled stockpiles, the “just-in-time” principle. When established approval routines were not followed, there was uncertainty about if externally donated PPEs, medical equipment, and other consumables could be used. Staff shortages were partly attributed to employees who struggled with the increased tempo. Care capacity suffered due to a lack of dedicated wards and beds.

### Barriers to response development

#### Anticipation difficulties

Anticipating the size of the pandemic was challenging as prognoses were often incorrect. ED and ICU capacity was inadequate, and difficult to rapidly expand, which created challenges in patient flow logistics, patient hand-offs, and increased risks as patient transports became necessary when capacity was exceeded. Staff uncertainty and unfamiliarity with a new disease, its symptoms, and diagnostic testing made it difficult to adapt treatment and care strategies to patient needs. Resource scarcity limited testing to those with clear symptomatology.

#### Crisis leadership

Crisis leadership was faulted for conflicting and rapidly changing directives from regional managers that often did not match the reality on the floor. Guideline discrepancies created communication challenges for managers as they struggled to inform staff. It also generated values conflicts, described as a tension between instructions and the professional ethos. Participants described the emotional difficulties of enforcing visitation restrictions on family members of dying patients.

When the instructions you receive do not match your values, you must eventually let go of the instructions. (P03)

#### Task shifting and facility repurposing

Task shifting and facility repurposing created new challenges. Participants’ clinical tasks increased, facility management demanded attention, there were concerns about losing well-functioning routines and units, and difficulties to distribute the large influx of external staff where they were needed most. Repurposed staff could therefore find themselves in a disorganized environment unable to train or support them adequately. Lack of knowledge about organizational structure and function under normal conditions slowed the transition from normal to crisis organization.

### Emerging consequences

Participants described how poor planning led to local stockpiling, displacement of other care needs, and staff brittleness. Hospitals feared an impending lack of resources, so staff hoarded equipment locally and did not send material to central stockpiles for redistribution to where it was needed most. Other care needs, e.g. non-acute surgery and check-ups were postponed, and participants expressed concern over a mounting “care debt” as patients seeking care for non-covid related conditions diminished. The pandemic strained the endurance and perseverance of staff that worked in a high state of readiness for months.

Our staff has not volunteered for this. It is so stressful, that we rationalize it like this: It is ok to cry on your way home from work– that is a natural reaction to an unnatural situation. However, it is not ok that staff cry when they come into work. (P03)

### New collaborations and support networks

While individuals’ frontline efforts in the microsystem around patients were in focus, participants also described collaboration at the meso (e.g. within and between hospitals) and macro levels (e.g. regional and national government agencies and international networks) that influenced the microsystem response.

### Learning collaborations

Hospitals contacted each other through their clinical training centers and linked with universities and colleges to share experiences, curricular design, so medical and naprapathy students could ward patients.

### Support for equipment, planning, and staffing

External actors repurposed and redirected staff, resources, and material to hospitals. Hospitals had daily contact with Swedish government, military, and ministries, the Swedish Association for Local Authorities and Regions, the National Board of Health and Welfare, and the Medical Products Agency to address the “enormous shortages” and resupply logistics. The Swedish work environment authority and the Ministry of development were vital in sourcing PPEs. Regionally, the Local Health Care Crisis Command (Lokal Särskild Sjukvårds Ledning, LSSL) assisted with ICU resource management, contingency planning, and taking competency inventories. Contacts were established with colleagues in other countries, such as Italy, China, and Taiwan to learn from their experiences.

### Resource reprioritization, repurposing, and redirecting

Responses to the challenges posed by the pandemic were associated with resource management, process management, and communications and relational capacity.

#### Resource management

Resource management included the management of system inputs through governance methods, decision-making, and competency exchange and training. Participants described resource management responses (e.g. PPEs, staffing, and competencies) at the macro, meso, and individual levels. On the national level, government ministries repaired logistics chains based on inventory information from hospitals. The media was described as an important ally, as it often brought more attention from decisionmakers than traditional communication channels.

Regionally, a temporary Stockholm Command Center was established working out of a hospital CEO’s office, collected logistics and medical competencies, identified needs, and together with companies such as Scania (truck manufacturer), SAS (airline), Coor (facility management), H&M (clothing retail company), Camfil (advance filter producer), and IKEA (furniture company) sourced PPE. Companies retooled to manufacture products needed by hospitals. Resource management attained greater clinical relevance when Health Care Services Stockholm County (SLSO) was given crisis management responsibility for the entire healthcare system.

At the organizational level, hospitals adapted to a high state of readiness with the ambition to always be one step ahead.

We have worked based on a data model of what we thought would happen… You cannot manage today based on how it looks today. You must manage based on how it will look in two weeks if you want to have a hope about being able to do something about it… you must always be one step ahead. (P11)

Hospitals increased ED staffing to handle the massive patient flows, extended shifts, and set new routines. Competency exchange and training occurred both internally within hospitals, and externally within the region and internationally. Doctors and nurses from other departments were given rapid ICU training and medical, nursing, and naprapathy students were trained and employed. International colleagues were invited to share their knowledge and experiences. Certain equipment was prioritized, particularly PPE and medicines, and stockpiles were created. Digital matrices were developed to connect managers with the resources they needed.

At the individual level, staff at some hospitals were scheduled for 12-hour shifts to meet patient demand and urged to plan patient time carefully so they could use scarce PPE for longer, but fewer, periods.

#### Process management

New PMs and routines, restructuring of flows and physical layout, restructuring of operations to increase capacity, down-prioritization of non-essential education, managing psychological factors, redirecting ambulances, support from external actors, and fast iterations.

The pandemic forced hospitals to reprioritize, repurpose, redirect, establish new routines, and innovate. ICU capacity was prioritized and rapidly increased within days, with space repurposed from other units. Non-essential education such as clinical skills training for medical students was down prioritized in favor of training staff for ICU service. Ambulance communication lines were improved to alert about patient arrivals or when patients were redirected. ED patient flows were restructured to repurpose space and staff for Covid-care. New routines were established for dealing with family and informal caregivers; digital tablets enabled patients to communicate with family and digital patient consultations increased dramatically. Participants described a constant pressure to move fast. Some ideas with potential were lost due to the lack of time to develop a wider understanding, acceptance, or support. Nevertheless, despite the pressure, there was a common understanding within organizations to approach problem-solving and innovation scientifically, i.e. not just haphazardly throw together a solution, but to proceed in a systematic manner grounded in scientific knowledge, evidence, and proven experience.

#### Communication and relational capacity

Participants described collaborations with other providers, working within a medical framework, establishing routines for dealing with family and informal caregivers, quicker communication lines, and more efficient digital meetings.

Close collaboration between units resulted in a better understanding of each other’s competencies and needs. Hospitals and the regional administration mitigated staff burnout by putting hearts on a wall to celebrate each discharged patient or arranging for ministers and psychologists to walk the floors. A knowledge acquisition group met regularly to coalesce lessons learned into practical guidelines that could be disseminated. Participants stressed that CEO’s medical expertise enabled them to understand and support managers.

Efforts to improve communication included an information hotline for citizens. Reporting routines upwards from the floor were improved, but some initiatives, such as a staff suggestions email, failed due to lack of awareness. The high threshold for digital meetings prior to the pandemic was overcome as everyone became better at using the technology.

### Organizational and individual factors influenced responses

Agile responses to the challenges were influenced by organizational facilitators, organizational barriers, and individuals’ desire to do good.

#### Organizational facilitators

Latent organizational factors that enabled a quick response included existing collaborations between units and hospitals, established catastrophe plans facilitated adaption of new guidelines, and existing digital health solutions were repurposed. Clinical educators quickly grasped the need for competency development of incoming, repurposed staff. Staff shared a common purpose and goal to solve the crisis, maintained a positive attitude, a desire to do good, and a willingness to make personal sacrifices.

#### Organizational barriers

The pandemic revealed serious difficulties in achieving collaboration across existing organizational boundaries, particularly between hospitals and primary care. Participants explained this was due to historical divides with poor communication and coordination, which made assigning responsibility for individual patients or patient segments such as non-hospitalized Covid-19 patients difficult. Inadequately formulated contracts with suppliers and the “just-in-time” principle were faulted by participants for frail supply-chains.

#### Individuals’ desire to do good

Participants described a common desire to make the most of one’s knowledge by sharing it with others, to learn and improve care delivery, and to do good. They saw widespread positive thinking and fighting spirit, and an optimism that individual action would lead to positive outcomes. They also described the eventual toll of sacrifices made, long shifts, delayed vacations, and skipped breaks.

### Collaboration, adaptation, and leadership were key

Lessons were learned related to collaboration, adaptive responses, and crisis leadership.

#### Systems-based collaboration

The pandemic highlighted the importance of effective collaboration with key partners and the wider system. Centrally placed decision-makers lacked the requisite knowledge and holistic perspective to enable this. Instead, hospitals developed their own strategies, and the university hospital took the lead in developing patient treatment strategies and acted as a safety net when other hospitals’ ICUs were filled. Hospitals with good results shared their strategies with the region and eventually the rest of Sweden as cases increased in other parts of the country.

#### Adapt responses

Hospitals chose to work systematically with a two-week time frame that allowed them to be agile and continually adapt their responses. Participants explained how they learned to mobilize quickly, without analyzing or dwelling on single activities for too long. In preparation for a future pandemic, participants emphasized the need to link organizational change with the quick response, shorten decision-making pathways, align crisis leadership, and establish plans for protracted health crises of magnitude.

#### Crisis leadership

Participants argued for a professions-led and distributed agile response to decision-making.

The management of health care usually ends up at the wrong level… Some have tried to control care from a central regional function… from there no one has an idea what it looks like in the ED… How can one manage from that place? It is absolutely impossible. This is why we end up in some weird situations when a strange directive comes from there. We have something to learn from this: the operational management of health care must be the purview of those who provide it. (P11)

Centralized support functions were essential– but in a support and not a control capacity.

That was the point of distributing crisis leadership to practitioners because then it became more relevant crisis leadership. (P08)

In the early stages, health care was stuck in administrative uncertainty and bureaucratic limbo as ministries negotiated questions of ownership, preventing support from external actors. In contrast, countries such as Norway, Denmark, and Spain established air bridges, which enabled companies to transport equipment.

## Analysis

The main challenges that emerged in the Swedish context were similar to the experience of many other countries during the first Covid-19 pandemic: Lack of pandemic preparation that led to acute socio-technical resource shortages (equipment, tools, skills, routines, and processes) and a need to rapidly adapt. The pandemic response engaged actors in new collaborations, raised awareness of the importance of initiatives to support psychological well-being, established new and improved existing communication channels, and lowered the threshold for implementation of digital tools. Responses involved reprioritization, repurposing, and redirecting resources; new routines; and innovation. Individual and organizational factors impacted these responses. Collaboration, adaptation, and leadership were key factors in these responses.

Table 2 presents a categorization of the empirical findings. The attributes which contributed to resilience occurred primarily at the situational and structural levels.

**Table 2:** Integrated Resilience Attributes Framework analysis [20] of the managerial and organizational characteristics that contributed (+) or limited (-) resilience over time throughout the health system

Resilience potentials	Situated resilience (micro)	Structural resilience (meso)	Systemic resilience (macro)
**Anticipating**		(+) Learning collaborations with internal actors, clinical training centers, universities and international contacts	(-) Lack of preparedness for a viral pandemic (-) Acute shortages (PPE, equipment, staff, capacity) (-) Difficult to anticipate pandemic volume
**Monitoring**		(+) Resource management	
**Responding**	(+) Infection control routines (-) Fear of lack of resources (-) Unfamiliarity with new products (-) Continued presence of Covid-19 (-) Medical challenges (-) Increased care needs, especially with older patients (-) Displacement of other care needs (+) Resource management (+) Restrict visitation (+) Staff shared a common purpose and goals (+) Staff attitudes (+) Interprofessional collaboration (+) Individuals’ desire to do good	(+) Task shifting and facility repurposing (+) Improved patient capacity (+) Support for equipment, staffing, planning (e.g. repurpose and redirect support to hospitals, collaboration with government agencies) (+) Communication and relational capacity (+) New PMs and routines (+) Restructure flows and operations (+) Focused, needs based competency training (+) Competency exchange (+) Down-prioritize non-essential education (+) Manage psychological factors (-) Historical organizational divides (-) Just-in-time supply model	(-) Transition from normal to crisis, and then back (-) Communication challenges (-) Conflicting and changing directives and guidelines (+) Governance methods and decision-making (-) Inflexible digital infrastructure (-) Suboptimal crisis leadership (-) Patient flow logistics including hand-offs
**Learning**	(-) Brittleness	(+) Become agile through fast iterations and quick mobilization with short decision pathways (+) Integrate organizational change	(+) System-wide collaboration (+) Share learnings (+) Pandemic response planning (+) Find balance between bureaucratic control and decentralized professional bureaucracies

The clear failures to *anticipate* (Row 1) the scope of the challenges and the stress this placed on staff in the frontline suggests that the pandemic exposed latent systemic shortcomings and exacerbated existing weaknesses. Hospitals and the health system were unprepared for the volume of patients and the protracted nature of the pandemic, which exceeded the predictions of existing crisis plans focused on “modern” threats of natural or man-made catastrophes. This lack of preparedness can be partly linked to system level failures to learn due to organizational divides between hospitals and primary care, a reliance on just-in-time production planning, and a failure to act on the existing knowledge of pandemic threats.

*Responding* (Row 3) efforts were primarily situated and structural (Columns 1 and 2). We identified a pattern of innovative actions that occurred simultaneously and at several levels of the system with multiple actors, *foci*, and communication lines. United by a common purpose, a tangible sense of urgency, and a desire to do good and make personal sacrifices, decentralized initiatives were taken on the frontlines, illustrating situated resilience.

Over time, when initial crises and contingency plans were exhausted, structural resilience was observed, such as when structures were developed to centralize decision-making. Centralized directives worked well when they were aligned with needs identified through individual and organizational initiatives, such as acquiring PPE. This alignment occurred when there was a concerted effort to *monitor* and understand the situation on the floor, to shorten communication lines, and when actors removed from the floor actively sought out information. A clinical background among decision-makers was described as conducive to these endeavors that involved the restructuring and reorganizing of practices. However, monitoring was not enough due to the volatility of the situation, instead, leaders needed to anticipate where they should be in two weeks’ time in order to be adequately prepared. This raises questions of the value of monitoring if it is done without anticipation.

As participants described, a tension developed between centralized command-and-control planning and decentralized professional bureaucracies that led to a moral stress where professional values eventually trumped centralized directives when centralized decisions or recommendations did not fit with the experienced reality of staff and managers in the thick of things.

The shortcomings experienced during *anticipating*, are indicative of poor previous systemic *learning*. The learnings at the systemic level can potentially contribute to a more resilient response in the future, but the lack of previous learnings hindered anticipation and placed undue stress on the microsystem. Systemic learning if maintained, could improve the anticipatory resilience for the next crises.

## Discussion

Region Stockholm, its health care organizations, and their employees, demonstrated facets of organizational resilience. Contributions to resilience primarily involved *responding* at the *situated* and *structural* levels, which required individual sacrifices, resource management, process management, and communications and relational capacity. The lack of *systemic* resilience, i.e. historical shortcomings in *learning* at the macro level, negatively impacted the *anticipatory* phase and potentially placed undue pressure on the micro (situated) and meso (structural) levels ´than would have been demanded in a more learning oriented health system.

Poor anticipation led to an initial inability and subsequent delay in the system to grasp the situation despite the signals coming from outside Sweden. Historically, pandemics are characterized by misinformation and denial [33]. And incorrect prognoses regarding patient volumes were indicative of maintaining linear thinking despite the complex challenge [34].

Historically, Sweden had well-developed contingency plans during the Cold War. These included large stockpiles of PPE eventually dismantled in the 1990s in the swells of Perestroika and Glasnost and the increased interconnectedness of the world made future large-scale military conflicts unthinkable [35]. Ironically, this interconnectedness made the reacquisition of material goods difficult as nations, organizations, and individuals began competing on a global scale against each other to acquire PPE. Some participants blamed “Just-In-Time” production planning that came with previous Lean initiatives. In contrast, The Mayo clinic used scenario planning to build upon medical, epidemiological, and historical knowledge to anticipate the need to quickly ramp up production capacity and in 2018 established new procurement practices as an alternative to stockpiling [36–38].

Effective responses were enabled by a common purpose, i.e. a shared mental model and prerequisite for self-organization [39]. Staff developed innovative responses motivated by this common purpose in a web of inventiveness with rapid adoption, exemplifying situated resilience. When existing crisis guidelines were found to be inadequate, and in the absence of an expected centralized response, physicians exemplified situated resilience by connecting with colleagues in Southern Europe to learn how to prepare [40]. This echoes similar findings of the preponderance of situated and structural resilience in lieu of systemic resilience [41]. The adoption threshold for existing digital health solutions and faster communication (digital meetings) appeared lower, suggesting that chaotic and urgently challenging situations like the Covid-19 pandemic could open people up to novel solutions that had previously been met with resistance, such as digital meetings and digital care, when they were viewed in the context of overcoming constraints [41–43].

The moral stress experienced by staff mirrors findings in another Swedish study, which found three managerial approaches in response to the pandemic: top down (managers make decisions without physician involvement), bottom-up (physicians mandated by managers to solve challenges), and grassroots (physicians initiate and carry-through response without managerial mandate or support) [40]. All three were experienced by physicians as either troublesome or extremely troublesome [40]. Lower levels of trust and confidence in centralized efficacy resulted in local stockpiling of resources and ad hoc local initiatives. Over the long term, this mismatch can discredit and weaken leaders’ credibility and influence [44].

Crisis leadership often includes an approach to stabilize through “clear” or “strong” leadership [45]. Under high pressure, with short timeframes and many unknowns (known and unknown), managers can be tempted to choose a “command-and-control” authoritarian response [46]. Participants described that this centralized decision-making, while a hallmark of catastrophe management planning in the region, proved inadequate to deal with the protracted timeframe of the first wave and the volume of response efforts. Instead, when things become complex, decentralized decision-making and placing the onus of responsibility for innovating response strategies with those best able to grasp the challenge and develop a response, i.e. frontline staff, is a more effective strategy [30, 47]. Leaders should therefore work from their clarity of purpose, expressed as a “commander’s intent”, prioritize between suggestions, and facilitate implementation by managing context instead of content [47, 48]. Such a response could potentially have allowed more innovative ideas to survive, as reflected on by study participants.

Leading without anticipatory resilience suggests that the examples of organizational resilience identified were less proactive and more defensive in nature and characterized by short-term thinking [25]. Increasing the learning capability of organizations could engender a longer-term perspective among leaders, which could improve the sustainability of innovative countermeasures developed during the pandemic and counteract the backslide that has already begun [15]. Predictive planning, simulations, scenario planning, and an infinite mindset are approaches that could improve systemic resilience [36, 49]. Future research could explore the value of dividing managers and staff into separate teams that focus on developing acute short-term or long-term responses in parallel, as suggested by Snowen and Boone [45]. Another could be to explore the impact on systemic learning to improve anticipatory resilience by coaching leaders to address short-term crises from an infinite mindset, i.e. a perspective that considers and preemptively accounts for the long-term consequences of short-term crisis problem-solving.

### Limitations

This study was conducted in the midst of the first wave of the pandemic, which we recognize created three main limitations. Most noticeable was the impact on data collection, as the increase workload limited accessibility to presumptive participants. The authors were also impacted in terms of personal health and response engagement, which could lead to biases. Therefore, to improve trustworthiness, we worked to ensure robust data collection and analysis processes that engaged all the authors. Through purposive sampling and snowballing, we sought to include the perspectives of study participants who were key leaders with influence over response development. In terms of analysis, we sought to eliminate biases through reflexive discussions among authors and a two-step methodological process that began with a “clean slate” of a primary inductive analysis followed with a directed content analysis to logically infer explanations for the findings with the help of an established resilience framework. Thirdly, the timeframe limited our ability to draw conclusions on the learning stage of resilience that could impact the anticipatory stage. Future studies are warranted to explore if and how learning processes developed within the studied organizations that led to adoption of new norms and practices.

## Conclusion

This study describes how the pandemic tested the resilience of a regional health care system, with implications beyond this particular system. Individuals and organizations repeatedly displayed situational and structural resilience in their responses. However, monitoring proved challenging and systemic resilience was hindered because initial systemic responses were experienced as counterproductive, and the lack of systemic learning hindered the anticipatory resilience potential. This placed an undue level of pressure on individuals and microsystems to respond, especially when centralized management and directives were misaligned with frontline realities and needs. Resilient “heroes” emerged as individuals and organizations stepped up. However, if health systems are able to improve their learning capabilities, perhaps we will need fewer heroes because the anticipatory and response resilience potentials will be that much higher.

### Electronic supplementary material

Below is the link to the electronic supplementary material.

Supplementary Material 1

Supplementary Material 2

## Data Availability

The dataset supporting the conclusions of this article is included within the article and its additional files.
