# Supplementary material for: Building resilience: analysis of health care leaders’ perspectives on the Covid-19 response in Region Stockholm
Source: BMC Health Serv Res. 2024 Apr 2;24:408. doi: 10.1186/s12913-024-10886-4 (PMC10985875; doi:10.1186/s12913-024-10886-4)
Supplement: Supplementary file 1 — Supplementary Material 1 [file 12913_2024_10886_MOESM1_ESM.docx]

**Additional File 1. The Interview Guide**

1. Please give a short presentation of who you are, what your current position is and what organization you represent.
2. What were the biggest challenges within your organization due to COVID-19?
3. Were there any unconventional methods that you applied?
4. Did you see any other possibilities in developing/changing areas of your organization because of COVID-19?
5. Could you tell how the pandemic played out according to you?
6. What were the most important goals?
7. What did you manage to achieve?
8. What circumstances contributed to positive results?
9. Thinking back, were there any game changers?
10. Did you cooperate with actors/organizations which you normally do not?
11. How were resources made available?
12. What have you learned thus far?
13. What weaknesses would you say have been exposed in the Swedish health care system?
14. What do you personally take away from the pandemic?
