# Supplementary material for: Building resilience: analysis of health care leaders’ perspectives on the Covid-19 response in Region Stockholm
Source: BMC Health Serv Res. 2024 Apr 2;24:408. doi: 10.1186/s12913-024-10886-4 (PMC10985875; doi:10.1186/s12913-024-10886-4)
Supplement: Supplementary file 2 — Supplementary Material 2 [file 12913_2024_10886_MOESM2_ESM.docx]

**Additional File 2. Themes, categories, and subcategories**

| Themes | Category | Subcategory |
| --- | --- | --- |
| Poor preparation led to acute shortages | Pandemic preparation | Lack of preparedness for a viral pandemic  Infection control routines  Unfamiliarity with new products  Inflexible digital infrastructure  Suboptimal crisis leadership |
|  |  |  |
|  |  |  |
|  | Acute shortages | Personal protective equipment  Medical equipment  Staff shortages  Care capacity |
|  |  |  |
|  | Anticipating pandemic development | Difficult to anticipate pandemic volume  Task shifting and facility repurposing  Improved patient capacity  Patient flow logistics including hand-offs  Medical challenges of patient care  Increased care needs, especially for older patients  Transition from normal to crisis organization (and then back)  Restrict visitation  Communication challenges  Conflicting and changing directives and guidelines  Continued presence of Covid-19  Brittleness |
|  |  |  |
|  |  |  |
|  |  |  |
|  |  |  |
|  |  |  |
|  |  |  |
|  |  |  |
|  |  |  |
|  |  |  |
|  |  |  |
|  |  |  |
|  |  |  |
|  |  |  |
|  | Emergence of organizational and social debt | Displacement of other care needs  Fear of lack of resources |
|  |  |  |
| New Collaborations and support networks | Learning collaborations | Internal actors  Clinical training centers  University  International contacts |
|  |  |  |
|  | Support for equipment, planning, and staffing | External actors repurposed and redirected support to hospitals |
|  |  | Collaborations with government agencies |
|  |  | Regional support for ICU resource management, contingency planning, and competency inventories |
| Resource Reprioritization, repurposing, redirecting | Resource management | Governance methods and decision-making  Competency exchange  Focused, needs-based competency training  Resource management |
|  |  |  |
|  | Process management | New PMs and routines  Restructure flows and operations  Down prioritize non-essential education  Manage psychological factors |
|  | Communication and relational capacity | Collaboration with other providers  Work within a medical framework  Establish routines for dealing with family and informal caregivers  Quicker communication lines  More efficient digital meetings  Response failure |
| Organizational and individual factors influenced responses | Organizational facilitators | Latent factors that enabled a quick response  Latent collaborative possibilities in the broader health care context  Identified competency development needs early  Staff shared a common purpose and goals  Staff attitudes |
|  |  | Interprofessional collaboration |
|  |  |  |
|  | Organizational barriers | Historical organizational divides  “Just-in-time” supply model |
|  |  |  |
|  | Individuals’ desire to do good | Share knowledge  Do good, be good  Personal sacrifice |
|  |  |  |
| Collaboration, Adaptation, and Leadership were Key | System-based collaboration | System-wide collaboration  Share learnings |
|  |  |  |
|  | Adapting responses | Become agile through fast iterations and quick mobilization with short decision pathways  Integrate organizational change  Pandemic response planning |
|  |  |  |
|  |  |  |
|  | Crisis leadership | Centralized bureaucratic control vs. decentralized professional bureaucracy |
